# Acetylcholine deficit causes dysfunctional inhibitory control in an aging-dependent manner

**DOI:** 10.1038/s41598-022-25402-z

**Published:** 2022-12-03

**Authors:** Paul Rafael Sabandal, Erick Benjamin Saldes, Kyung-An Han

**Affiliations:** Department of Biological Sciences, University of Texas at El Paso, El Paso, TX 79968 USA

**Keywords:** Motor control, Neural ageing

## Abstract

Inhibitory control is a key executive function that limits unnecessary thoughts and actions, enabling an organism to appropriately execute goal-driven behaviors. The efficiency of this inhibitory capacity declines with normal aging or in neurodegenerative dementias similar to memory or other cognitive functions. Acetylcholine signaling is crucial for executive function and also diminishes with aging. Acetylcholine’s contribution to the aging- or dementia-related decline in inhibitory control, however, remains elusive. We addressed this in *Drosophila* using a Go/No-Go task that measures inhibition capacity. Here, we report that inhibition capacity declines with aging in wild-type flies, which is mitigated by lessening acetylcholine breakdown and augmented by reducing acetylcholine biosynthesis. We identified the mushroom body (MB) γ neurons as a chief neural site for acetylcholine’s contribution to the aging-associated inhibitory control deficit. In addition, we found that the MB output neurons MBON-γ2α’1 having dendrites at the MB γ2 and α’1 lobes and axons projecting to the superior medial protocerebrum and the crepine is critical for sustained movement suppression per se. This study reveals, for the first time, the central role of acetylcholine in the aging-associated loss of inhibitory control and provides a framework for further mechanistic studies.

## Introduction

Acetylcholine is important for executive function in human subjects and rodents. In healthy non-smokers, for example, acute nicotine administration enhances attention when tested in the spatial attentional resource allocation and rapid visual information processing tasks^1^. Consistently, the nicotinic acetylcholine receptor (nAChR) antagonist mecamylamine administration in rats and the nAChR beta2 knockout in mice impair attention performance in the 5-choice serial reaction time task (5-CSRTT)^2,3^, substantiating a crucial role of acetylcholine in attention. The role of acetylcholine in inhibitory control, however, seems rather complex. In healthy non-smokers, acute nicotine administration improves behavioral inhibition in the stop signal task^4^. Nicotine treatment in rats, on the other hand, impairs inhibitory action and choice when tested in the 5- or 3-CSRTT, go/no-go task and systemic delayed reward task^2,5,6^. The inhibitory effect of nicotine is blocked by mecamylamine pretreatment^6^ or the infusion of the alpha4beta2 nAChR antagonist dihydro-beta-erythroidine (DHbetaE) in the infralimbic cortex^5^. Moreover, the DHbetaE infusion^5^ or systemic mecamylamine administration^2^ without nicotine administration is sufficient to suppress impulsive action, suggesting pro-impulsive function of endogenous acetylcholine in rats, which is not in line with the findings in human subjects. It seems of great value to study an additional model organism for deeper understanding of the mechanism by which acetylcholine regulates inhibitory response control.

Acetylcholine neurotransmission declines with normal aging and the decline is highly augmented in Alzheimer’s disease and related dementias such as frontotemporal dementia and Lewy body dementia^7–11^. Executive dysfunctions, in particular impaired inhibitory control, represent prominent symptoms of early-stage Alzheimer’s disease, frontotemporal dementia and other dementias^12–16^; however, there is no information on the role of acetylcholine in inhibitory control dysfunction associated with normal aging or dementias. To address this knowledge gap, we developed a simple behavioral paradigm to study a fundamental form of inhibitory response control in *Drosophila melanogaster*. Here, we show that acetylcholine significantly contributes to the aging-dependent deficit in inhibitory control and its major neural site is the mushroom body neurons.

## Materials and methods

### Fly strains and culture

The wild-type strain used in this study is *Canton-S* (*CS*). The following fly strains were obtained from the Bloomington Drosophila Stock Center (BDSC; Bloomington, IN): *Ace*^*c00215*^ (BDSC #10026), *ChAT*^*MI08244*^ (BDSC #55439), *ChAT*^*MI04508*^ (BDSC #37817), *OK107-GAL4* (BDSC #854), *MB010B-GAL4* (BDSC #68293), *201y-GAL4* (BDSC #4440), *MB009B-GAL4* (BDSC #68292), *c739-GAL4* (BDSC #7362), *MB008B-GAL4* (BDSC #68291), *c305a-GAL4* (BDSC #30829), *MB005B-GAL4* (BDSC #68306), *MB077B-GAL4* (BDSC #68283), *MB051B-GAL4* (BDSC #68275), *MB018B-GAL4* (BDSC #68296), *MB082C-GAL4* (BDSC #68286), *MB543B-GAL4* (BDSC #68335), *MB027B-GAL4* (BDSC #68301), *MB549C-GAL4* (BDSC #68373), *MB542B-GAL4* (BDSC #68372), *GH146-GAL4* (BDSC #30026) and *UAS-ChAT*^*RNAi*^ (BDSC #25856); *pBDP-GAL4* from David Anderson (California Institute of Technology, Pasadena, CA); *NP225-GAL4* from Andreas Thum (Universität Leipzig, Leipzig, Germany); *MB247-GAL4* from Scott Waddell (University of Oxford, Oxford, UK); and *NP1131-GAL4* from Josh Dubnau (Stony Brook University School of Medicine, Stony Brook, NY).

Flies were raised on a standard cornmeal/sucrose/yeast/agar medium at 25° C with 50% relative humidity under a 12 h light/12 h dark cycle. Flies were collected under carbon dioxide within two days after eclosion and were housed in mixed sex groups. For aging, all fly strains were transferred to fresh food every 2–3 day until the ages of 4 day, 2 week and 4 week. These ages were selected to examine flies at young (4 day), mid (2 week) and early old (4 week) ages. The 4-wk old allows capturing the brain and behavioral changes associated with early-stage dementia and potentially distinguishing them from those occurring during normal aging^17^. Prior to behavioral tests, flies were sorted into a group of 13 per food vial (representing n = 1) and were then left to rest for 2 day in the light-, temperature-, and humidity- controlled incubator before behavioral or immunohistochemical analyses. Both males and females were examined separately but there was no sex difference thus combined data are presented.

### Behavioral analysis

Flies were gently transferred into a rectangular plexiglass chamber (60 mm L × 60 mm W × 15 mm H) connected to filtered air and acclimated to the chamber for 10 min. The 10 L/min airflow was delivered to the chamber for 10 min. The chamber was video recorded to monitor fly movements before and after airflow. Videos were analyzed manually to score the number of flying events or using the Viewer3 tracking software (BiObserve Technologies, Bonn, Germany) that allows tracking and measuring the average speed of individual flies in mm/s per fly. Raw data were transported to Excel (Microsoft, Redmond, WA) and the number of the movements exceeding 60 mm/s (flying event) that we defined as loss of inhibition events (LIE) was scored per fly per min. To quantitively compare different conditions (e.g., age or genotype), we analyzed LIE numbers in 1 min bins for the entire 10 min of the No-Go phase and compared the 1 min bin having the highest LIE counts. All behavioral experiments were performed blind to the experimenter. The control and experimental groups were tested in the same experimental session in a randomized manner. Multiple independent sets of flies obtained from independent crosses were used for behavioral experiments.

### Immunohistochemical analysis

Immunostaining was performed as previously described^18,19^ with several modifications. Fly brains were dissected in ice-cold 1X phosphate buffered saline (1X PBS), fixed in 4% paraformaldehyde in 1X PBS for 3 h at 4 °C. The fixed brains were washed with 1X PBS once for 10 min, washed twice with 1X PBHT (20 mM PO_4_, 0.5 M NaCl, 0.2% Triton X-100, pH 7.4) for 10 min each, solubilized with 1% Triton X-100 in 1X PBHT for 1 h at room temperature, blocked with 5% normal goat serum (NGS) in 1X PBHT for 16–18 h at 4 °C and incubated with the 1X PBHT containing 1:1000 anti-ChAT antibody (ChAT 4B1^20^; DSHB, Iowa City, IA) and 5% NGS for 48 h at 4 °C. After two 10 min washes at room temperature and a 16–24 h wash at 4 °C with 1X PBHT, the brains were incubated with the Alexa Flour 488 conjugated goat anti-mouse IgG (A-11001; Invitrogen/Life Technologies, Eugene, OR) for 48 h at 4 °C followed by three 20 min washes with 1X PBHT and three 10 min washes with 0.12 M Tris–HCl, pH 7.2. The brains were then mounted in the Vectashield mounting medium (H-1000; Vector Laboratories, Burlingame, CA) for imaging with the 40X oil immersion objective in the LSM700 confocal microscope (Zeiss, Thornwood, NY). Pixel resolution was set at 2048 × 2048. Optical sections were made at 1 μm thickness and 50 sections were stacked for the representative images in Fig. 2B. ChAT immunoreactivity in the superior medial protocerebrum was quantified using the ImageJ (NIH, Bethesda, MD). The fluorescent signals were converted to grayscale and the fluorescence intensity per pixel in the SMP was calculated by [Integrated density_Total_/Area_Total_] per brain. The fold change of the fluorescent intensities in the *ChAT/*+ mutant brains from those in the control *Canton-S* brains was used for data presentation.

### Acetylcholine analysis

To measure acetylcholine, we utilized a commercially available Choline/Acetylcholine assay kit (catalog # ab65345, Abcam, Cambridge, United Kingdom). Briefly, 50 frozen fly heads per condition (genotype and age) were homogenized 50 μl of the Choline Assay Buffer (1 fly head per 1 μl) on ice with the KONTES Micro Tissue Grinder (Thermo Fisher Scientific, Waltham, MA) for 30 to 45 s. The homogenates were transferred to fresh tubes, centrifuged at 14,000 rpm for 10 min at 4 °C, and subjected to the Choline/Acetylcholine assay per the manufacture’s instruction. Multiple independent sets of fly heads (e.g., 4 to 8 sets) obtained from independent crosses were used for acetylcholine quantification.

### Statistical analysis

All statistical analyses were performed using the Minitab software (Minitab, State College, PA) or JMP (SAS, Cary, NC). Raw data were analyzed using the Anderson–Darling goodness-of-fit test for distribution and are reported as mean + SEM. Normally distributed data were analyzed by either two-tailed Student’s *t* test for two groups or by ANOVA followed by post hoc Tukey’s multiple-comparison for three or more groups or Dunnett’s test to compare experimental groups with a control group. Significant difference among the groups under comparison was determined using an α level of 0.05 in all analyses. All raw data files are available on request.

## Results

### Inhibitory control diminishes with age in *Drosophila*

**Figure 1:**
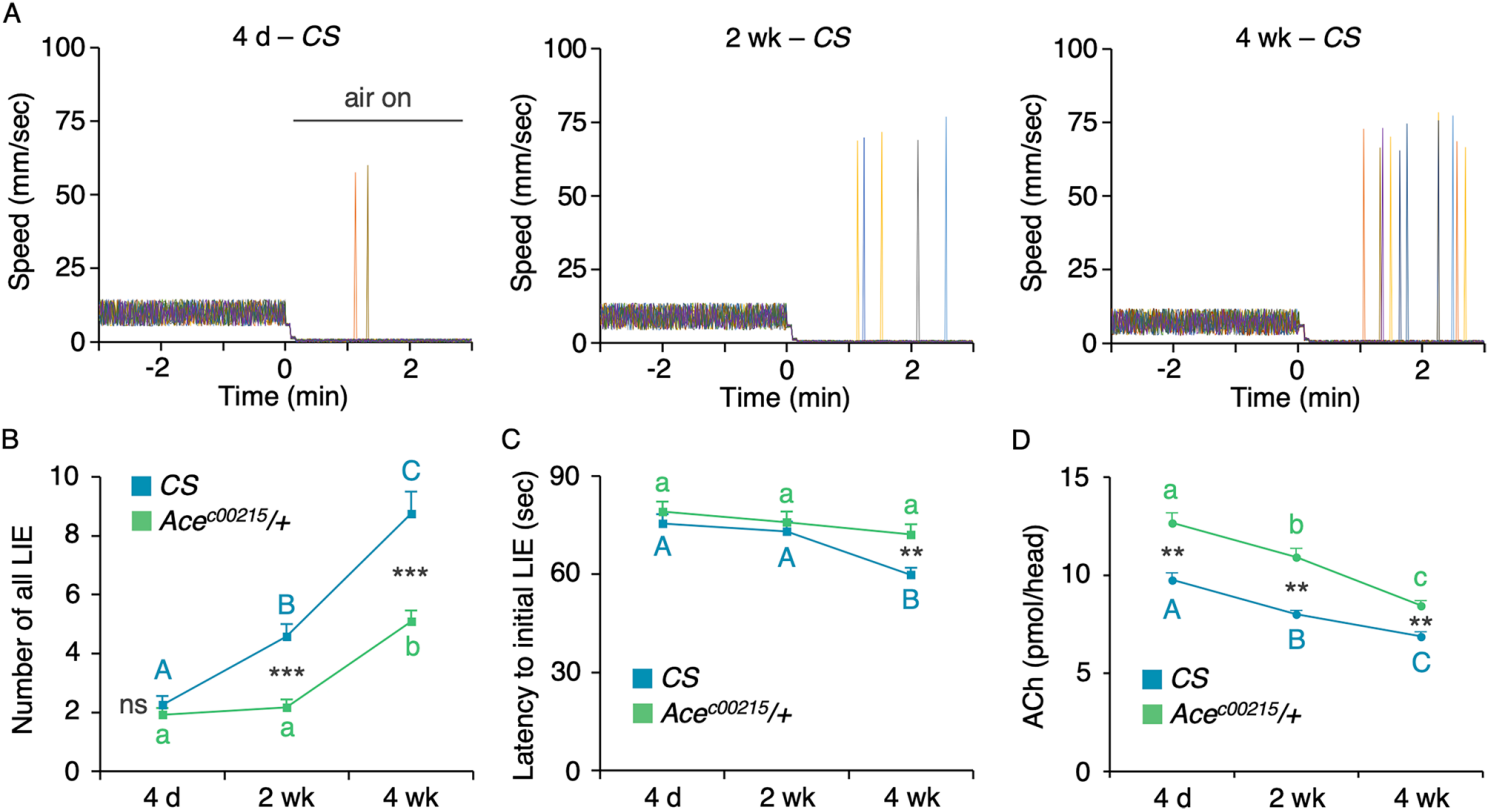
Aging diminishes inhibitory control in flies. (**A**) Representative movement traces of a group of 13 *CS* flies subjected to the GNG test in three chambers—one with the 4 day old, the other with the 2 week old and the last with the 4 week old. Each panel illustrates individual fly traces, which are denoted by the different colored lines. The timepoint when strong airflow was introduced is marked as 0 in the X axis. The *CS* flies of all three ages exhibited robust inhibitory control under airflow with infrequent instances of flying behavior representing LIE (movement > 60 mm/s). The sporadic LIEs increased with aging. (**B**) Quantitative representation of the aging-dependent increases in LIE and rescue by *Ace* deficiency**.** All LIEs displayed by 13 flies in a chamber for the entire 10 min under airflow during GNG test were manually counted revealing more frequent LIE in older *CS* flies (blue line). The aging-dependent increases in the LIE were alleviated by the heterozygous *acetylcholinesterase* (*Ace*) mutation (green line; *Ace*^*c00215*^*/*+) fully at the 2 week old and partly at the 4 week old. ANOVA with post hoc Tukey multiple comparison of three ages in each genotype: different letters (A, B and C for *CS*; a and b for *Ace*^*c00215*^*/*+) denote statistically significant differences (*p* < 0.05; *n* = 12). Student *t*-test between two genotypes (*Ace*^*c00215*^*/*+ and *CS*) in each age: ns, *p* > 0.05; ***, *p* < 0.0001; *n* = 12. (**C**) Latency to initial LIE. ANOVA with post hoc Tukey multiple comparison of three ages in each genotype: different letters (A and B for *CS*) denote statistically significant differences (*p* < 0.0001; *n* = 12). When the age-matching *CS* and *Ace*^*c00215*^*/*+ were compared by Student *t*-test, there is no difference in the LIE latency at 4 day or 2 week (*p* > 0.05, *n* = 12; not noted in the graph) but there is significant difference at 4 week (**, *p* < 0.005, *n* = 12). The heterozygous *Ace* mutation fully reversed the aging-related premature LIE latency. (**D**) Acetylcholine levels in the *CS* and *Ace*^*c00215*^*/*+ heads. The acetylcholine levels were significantly higher in *Ace*^*c00215*^*/*+ than *CS* at all ages and dampened with aging in both genotypes. ANOVA with post hoc Tukey multiple comparison of three ages in each genotype: different letters (A, B and C for *CS*; a, b and c for *Ace*^*c00215*^*/*+) denote statistically significant differences (*p* < 0.05; *n* = 4). Student *t*-test between two genotypes (*Ace*^*c00215*^*/*+ and *CS*) in each age: **, *p* < 0.005; *n* = 4.

Inhibitory control confers the ability to suppress inappropriate actions or thoughts and is a key executive function supporting flexible and goal-directed behaviors. This capacity declines with aging in human subjects, for example when tested in the go/no-go (GNG)^21^. A GNG task measures action restraint, requiring subjects to withhold a motor response to a “no-go” signal ^22^. To assess whether inhibitory control declines with aging in *Drosophila* similar to humans, we developed a *Drosophila* version of a GNG test, in which a group of 13 flies placed in a chamber were video-tracked to quantify their movements. Without salient stimuli, the wild-type *Canton-S* (*CS*) flies freely move around. Upon introduction of strong airflow, flies halt movements presumably to keep their behavior under control (Fig. 1A)^23,24^. The fly’s ability to suppress ongoing activity (walking) inappropriate in a given context (strong “wind”), and we postulate that it represents a fundamental form of inhibitory response control. We tested the female and male *CS* flies at three different ages 4 day (young), 2 week (mid), and 4 week (early old). Regardless sex and age, the *CS* flies exhibited robust movement suppression under strong airflow with infrequent instances of flying behavior, which is an impulsive act resulting from loss of movement inhibition (Fig. 1A). To identify whether the frequency of the loss of inhibition events (LIE) changes with aging, we counted the movement with the speed over 60 mm/s, consisting of flying. Notably, older *CS* flies exhibited more LIE (Fig. 1B: one way ANOVA; *R*^2^ = 0.704, *F*_*2,33*_ = 39.24, *p* < 0.0001; *n* = 12) at shorter latency for LIE (Fig. 1C: one way ANOVA; *R*^2^ = 0.372, *F*_*2,33*_ = 9.77, *p* = 0.0005), indicating that aging diminishes inhibitory capacity. We did not observe any difference in LIE between females and males at all ages thus the data obtained from both sexes are combined and presented in Fig. 1B and C.

It is known in human subjects and rodents that acetylcholine neurotransmission modestly declines with aging^7,8^ but this has not been demonstrated in flies. To test whether diminished acetylcholine neurotransmission is responsible for the aging-associated increase in LIE, we examined the *Ace*^*c00215*^*/*+ flies with the heterozygous mutation in acetylcholinesterase (Ace; EC 3.1.1.7) that breaks down acetylcholine and is a target for the first line medication to treat Alzheimer’s disease. The *Ace*^*c00215*^ allele is a severe hypomorph or null since it has a piggyBac insertion in the 5’ non-coding region of the gene and homozygous lethal^25^. We found that the heterozygous *Ace* mutation rescued fully at 2 week and partly at 4 week the movement suppression deficit (Fig. 1B: two-way ANOVA, *F*_*5,66*_ = 37.98, *p* < 0.0001; genotype effect, *p* < 0.0001; age effect, *p* < 0.0001; genotype × age, *p* = 0.0009) as well as the LIE latency at 4 week (Fig. 1C: two-way ANOVA, *F*_*5,66*_ = 5.236, *p* = 0.0004; genotype effect, *p* = 0.0112; age effect, *p* = 0.0007; genotype × age, *p* = 0.2021). Consistently, the *Ace*^*c00215*^*/*+ flies showed higher acetylcholine levels compared to *CS* at all ages tested and both genotypes have declining acetylcholine levels with aging (Fig. 1D: two-way ANOVA, *F*_*5,18*_ = 36.93, *p* < 0.0001; genotype effect, *p* < 0.0001; age effect, *p* < 0.0001; genotype × age, *p* = 0.116). This finding supports that diminished acetylcholine neurotransmission accounts for inhibitory control decline in normal aging, and the GNG test is well suited to study aging-related changes in inhibitory control in flies.

### Choline acetyltransferase (ChAT) deficit augments inhibitory control impairments with aging

**Figure 2:**
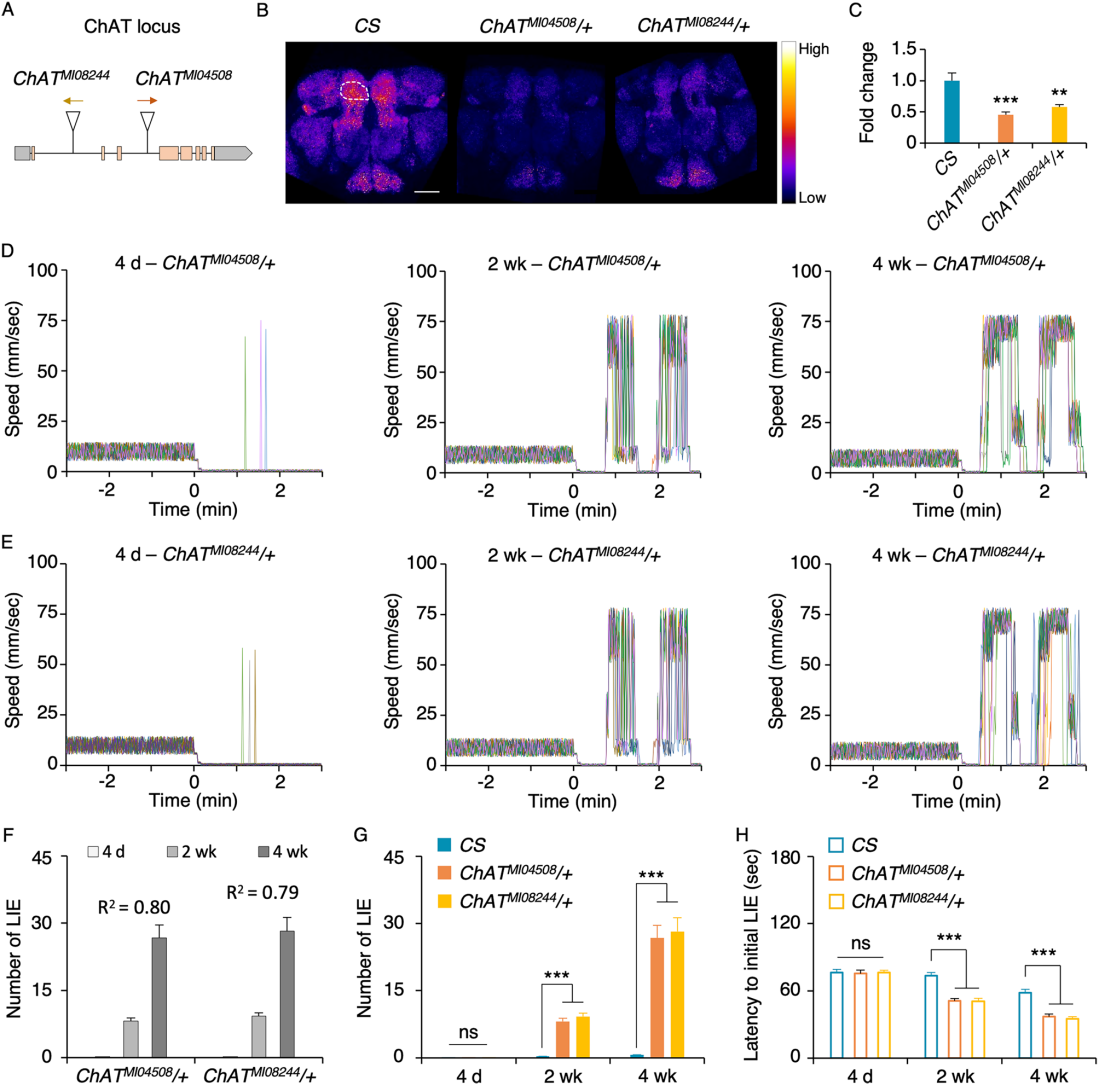
ChAT deficit amplifies the impaired inhibitory control with aging. **(A**) The scheme depicts the locations of the transposon MiMIC insertions in the *ChAT* locus: *ChAT*^*MI04508*^ (the orange arrow denoting direction of the splice acceptor in the transposon) and *ChAT*^*MI08244*^ (yellow arrow denoting direction of the splice acceptor). The coding regions are noted in orange boxes, noncoding regions in gray boxes, and introns in gray lines (not to scale). (**B**)ChAT immunoreactivity in the brains of *CS, ChAT*^*MI04508*^*/*+ and *ChAT*^*MI08244*^*/*+ . Signal intensity is converted into a fire scale. Quantification of ChAT immunoreactivity was done in the superior medial protocerebrum (SMP) in both hemispheres, one of which is demarcated with a white circle. Both heterozygous *ChAT* mutants had less ChAT immunoreactivity compared to *CS*. Scale bar, 50 μm. (**C**) Quantified ChAT immunoreactivity comparison. ChAT immunoreactivity levels were normalized with the average ChAT immunoreactivity level of *CS*. The heterozygous ChAT mutants showed significantly reduced ChAT immunoreactivity in the SMP compared to *CS* (ANOVA with post hoc Dunnett test using *CS* as a control: **, *p* = 0.0006; ***, *p* < 0.0001; *n* = 8–11). **(D,E**) Each graph depicts the representative movement traces of 13 flies in a chamber: *ChAT*^*MI04508*^*/*+ (**D**) and *ChAT*^*MI08244*^*/*+ (**E**) across different ages (4 day, 2 week, and 4 week). (**F,G**) Comparison of the number of LIEs per fly per min for *ChAT*^*MI04508*^*/*+ , *ChAT*^*MI08244*^*/*+ and *CS* at three different ages. The older heterozygous *ChAT* mutants exhibited higher LIEs (**F**: ANOVA *R*^2^ values are noted in the graph; *n* = 12), which are significantly more than those of *CS* at the ages of 2 week and 4 week but not at the age of 4 day (**G:** ANOVA with post hoc Dunnett test using *CS* as a control: ns, *p* > 0.05; ***, *p* < 0.0001; *n* = 12). (**H**) Latency to initial LIE. ANOVA with post hoc Dunnett using *CS* as a control: ns, *p* > 0.05; ***, *p* < 0.0001; *n* = 12). The heterozygous *ChAT* mutation caused significantly earlier onset of LIE at the ages of 2 week and 4 week.

To directly address the role of acetylcholine in inhibitory control, we used the flies defective in ChAT. ChAT (EC 2.3.1.6) is the biosynthetic enzyme for acetylcholine, which aids in the transfer of an acetyl group in Acetyl-CoA to choline, and *Drosophila* has a single gene for ChAT as in humans^25^. We examined two mutant alleles *ChAT*^*MI04508*^ and *ChAT*^*MI08244*^ containing the gene trap MiMIC vector^26^ (Fig. 2A). The homozygous *ChAT*^*MI04508*^ or *ChAT*^*MI08244*^ mutants die during development thus we investigated their heterozygous mutants. The ChAT immunoreactivity was widespread in the wild-type *CS* brain and its level was significantly reduced in both mutants at the age of 4 to 5 day old (Fig. 2B, representative images; 2C, immunoreactivity quantifications; ANOVA, *F*_*2,26*_ = 15.96, *p* < 0.0001, *n* = 8—11). When subjected to the GNG test, both heterozygous mutants showed dysfunctional movement suppression in an aging-dependent manner (Fig. 2D and E: the representative movement traces per chamber containing 13 flies of the *ChAT*^*MI04508*^*/*+ or *ChAT*^*MI08244*^*/*+ genotypes at the ages of 4 day, 2 week or 4 week). Specifically, the heterozygous *ChAT* mutants at all ages showed robust movement suppression when strong air was introduced (time 0 on the X-axis in Fig. 2D and E) similar to *CS*; however, they exhibited highly augmented LIEs as they got older (Fig. 2F: *F*_*2,33*_ = 65.28, *p* < 0.0001*, n* = 12 for *ChAT*^*MI04508*^*/*+ ; *F*_*2,33*_ = 61.56, *p* < 0.0001*, n* = 12 for *ChAT*^*MI08244*^*/*+). At the age of 4 day, there was no difference in LIEs of the *ChAT*^*MI04508*^*/*+ and *ChAT*^*MI08244*^*/*+ mutants compared to that of *CS* (Fig. 2G 4 day: *F*_*2,33*_ = 0.1377, *p* = 0.87*, n* = 12); however, there was significant increases in the LIEs of the *ChAT*^*MI04508*^*/*+ and *ChAT*^*MI08244*^*/*+ flies at the ages of 2 week (*F*_*2,33*_ = 65.35, *p* < 0.0001*, n* = 12) and 4 week (*F*_*2,33*_ = 41.3, *p* < 0.0001*, n* = 12) compared to those of *CS*. Also, the LIE onsets of *ChAT*^*MI04508*^*/*+ and *ChAT*^*MI08244*^*/*+ flies were significantly earlier than CS at 2 week (Fig. 2H; *F*_*2,33*_ = 26.996, *p* < 0.0001*, n* = 12) and 4 week (*F*_*2,33*_ = 32.365, *p* < 0.0001*, n* = 12) but not at 4 day (*F*_*2,33*_ = 0.0346, *p* = 0.97*, n* = 12). Together, these data indicate that the flies with reduced ChAT expression have augmented sensitivity to aging in movement suppression deficit, indicating the critical role of acetylcholine in the aging-dependent loss of inhibitory control.

### *Ace* deficiency rescues anomalous inhibitory control of the *ChAT* mutants

**Figure 3:**
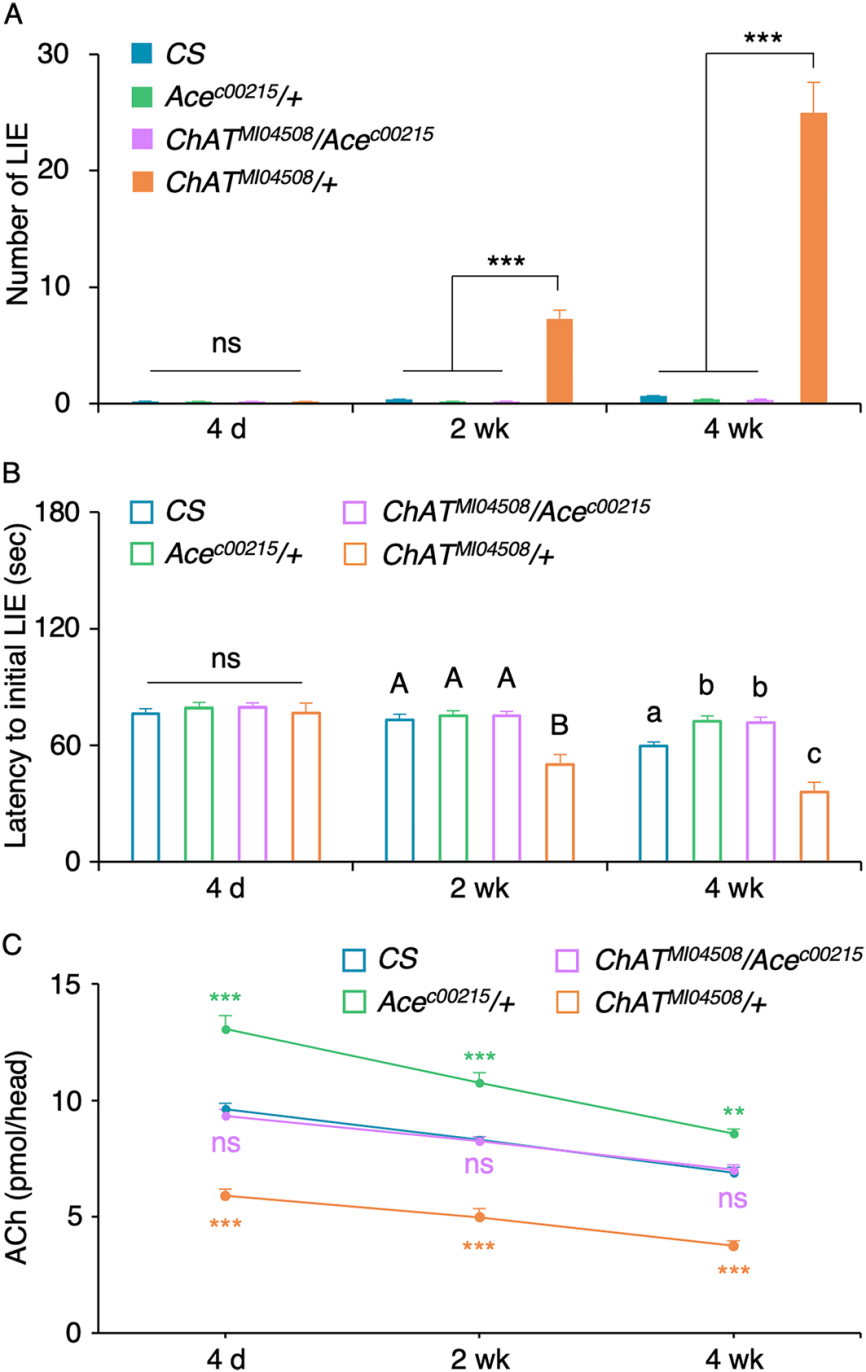
The heterozygous *Ace* mutation fully suppresses the *ChAT/*+ ’s inhibitory control deficits. **(A**) The double heterozygous *ChAT*^*MI04508*^*/Ace*^*c00215*^ (light purple) mutants displayed robust movement restraint with rarely detectable LIEs similar to *CS* (blue), *Ace*^*c00215*^*/*+ (light green) and *ChAT*^*MI04508*^*/*+ (orange) at the age of 4 day (one way ANOVA with post hoc Tukey: ns, *p* > 0.05; *n* = 12). At the ages of 2 week and 4 week, the *ChAT*^*MI04508*^*/Ace*^*c00215*^ mutants displayed a small increase in LIE similar to *CS* and *Ace*^*c00215*^*/*+ but the level was significantly different from that of *ChAT*^*MI04508*^*/*+ (ANOVA with post hoc Tukey: ***, *p* < 0.0001; *n* = 12). (**B**) The latency to the initial LIE was comparable in all genotypes under study at the age of 4 day (ANOVA with post hoc Tukey: ns, *p* > 0.05; *n* = 12). The double heterozygous *ChAT*^*MI04508*^*/Ace*^*c00215*^ exhibited the significantly delayed latency compared to that of *ChAT*^*MI04508*^*/*+ at the ages of 2 week and 4 week (ANOVA with post hoc Tukey: different letters denote statistically significant difference, *p* < 0.01, *n* = 12). (**C**) The acetylcholine levels in the double heterozygous *ChAT*^*MI04508*^*/Ace*^*c00215*^ were comparable to *CS*, but significantly lower than *Ace*^*c00215*^*/*+ and significantly higher than *ChAT*^*MI04508*^*/*+ at all ages. ANOVA with post hoc Tukey multiple comparison of the genotypes within an age: ns, *p* > 0.05; **, *p* < 0.005; ***, *p* < 0.0001; *n* = 4–8.

We reasoned that dampened cholinergic transmission causes the dysfunctional inhibitory control of the ChAT mutant. Should it be the case, we should be able to rescue the phenotype by reducing the amount of the acetylcholine degradation enzyme Ace. We tested this notion by examining the *ChAT/*+ mutant flies carrying the heterozygous *Ace* mutation^27^ (i.e. *ChAT*^*MI04508*^/*Ace*^*c00215*^) along with the flies with the heterozygous mutation only in *ChAT* or *Ace* and *CS* as controls. When subjected to the GNG test, there was no differences in LIEs of all genotypes at the age of 4 day as expected (Fig. 3A: ANOVA; *F*_*3,44*_ = 0.1621, *p* = 0.92*, n* = 12). Notably, the double heterozygous *ChAT*^*MI04508*^*/Ace*^*c00215*^ mutants exhibited the LIEs comparable to those of *CS* and *Ace*^*c00215*^/+ but significantly different from that of *ChAT*^*MI04508*^/+ at the ages of 2 week (Fig. 3A: ANOVA; *F*_*3,44*_ = 89.13, *p* < 0.0001*, n* = 12) and 4 week (*F*_*3,44*_ = 87.43, *p* < 0.0001*, n* = 12). Likewise, the double heterozygous *ChAT*^*MI04508*^*/Ace*^*c00215*^ mutants exhibited the latency to the first LIE comparable to those of *CS* and *Ace*^*c00215*^/+ but significantly delayed than that of *ChAT*^*MI04508*^/+ at the age of 2 week (Fig. 3B: ANOVA; *F*_*3,44*_ = 23.07, *p* < 0.0001*, n* = 12). At the age of 4 week, the *ChAT*^*MI04508*^*/Ace*^*c00215*^ mutants showed the latency comparable to that of *Ace*^*c00215*^/+ but significantly delayed than those of *ChAT*^*MI04508*^/+ as well as *CS* (*F*_*3,44*_ = 49.22, *p* < 0.0001*, n* = 12). Together, the heterozygous *Ace*^*c00215*^ mutation fully suppressed the *ChAT*^*MI04508*^*/*+ mutant’s LIE and latency phenotypes. In line with these results, the acetylcholine levels in the double heterozygous *ChAT*^*MI04508*^*/Ace*^*c00215*^ were indistinguishable from those of *CS* at all ages and they were significantly higher than those of *ChAT*^*MI04508*^*/*+ (Fig. 3C: ANOVA with post hoc Tukey multiple comparison of the genotypes in each age. For 4 day: *F*_*3,28*_ = 62.71, *p* < 0.0001; For 2 week: *F*_*3,12*_ = 63.26, *p* < 0.0001; For 4 week: *F*_*3,12*_ = 89.62, *p* < 0.0001; ns, *p* > 0.05; **, *p* < 0.005; ***, *p* < 0.0001; *n* = 4–8). This indicates that the acetylcholine deficiency is responsible for the aging-associated deficit in inhibitory control.

### The cholinergic neurons contributing to the aging-associated deficit in inhibitory control

**Figure 4:**
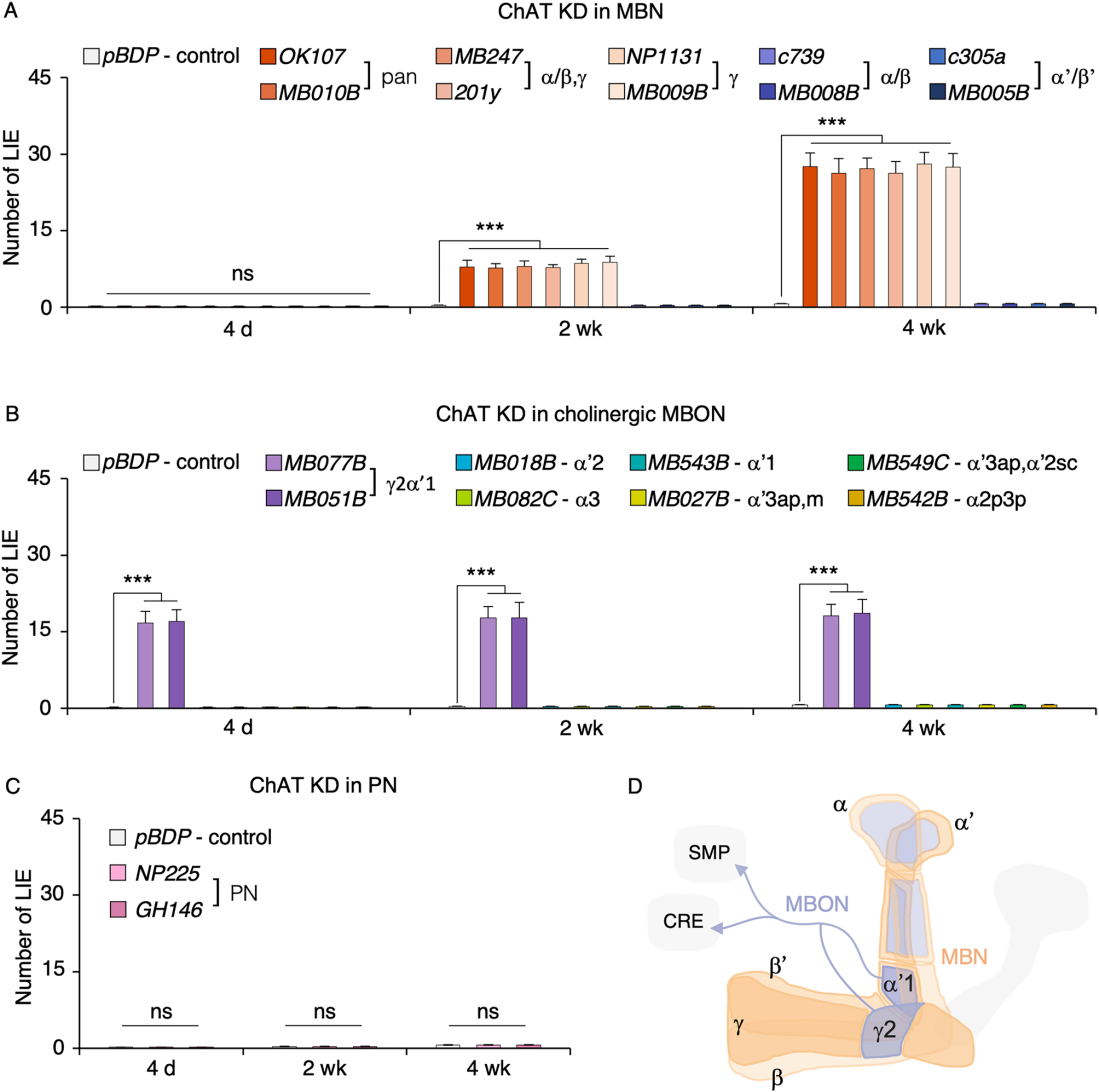
The cholinergic neurons important for inhibitory control. (**A**–**C**) To identify the cholinergic neurons involved in the aging-related LIEs, we knocked down ChAT by expressing *UAS-ChAT RNAi* in MBNs, MBONs or PNs. The promoter-less *pDBP*-GAL4 driver served as a control. (**A**) ChAT KD in either all-MBNs (via OK107-GAL4 or MB010B-GAL4), MBN α/β and γ (via MB247-GAL4 or 201y-GAL4), or MBN γ (via NP1131-GAL4 or MB009B-GAL4), but not in MBN α/β (via c739-GAL4 or MB008B-GAL4) or MBN α’/β’ (via c305a-GAL4 or MB005B-GAL4), led to the aging-dependent increase in LIEs (ANOVA with post hoc Dunnett test using *pDBP*-GAL4 as a control: ***, *p* < 0.0001; *n* = 8). (**B**) ChAT KD in the MBON-γ2α’1 (via MB077B or MB051B-GAL4) increased LIEs at all ages tested (***, *p* < 0.0001; *n* = 8). ChAT KD in other cholinergic MBONs (the GAL4 drivers used for KD and their dendritic sites are noted in the top panel) did not affect the flies’ movement inhibition (*n* = 8). (**C**) ChAT KD in the PNs (via NP225-GAL4 or GH146-GAL4) did not affect flies’ inhibitory control (ns, *p* > 0.05; *n* = 8). (**D**) Scheme of the cholinergic neurons important for inhibitory control that include the mushroom body neurons (MBNs; orange) and several mushroom body output neurons (MBONs; purple) as well as the PNs (not shown). The PNs have dendrites in the antenna lobe and project axons to the MBN calyx. The MBNs include γ, α/β and α’/β’ among which ChAT in γ (dark orange) is important for the aging-related LIEs. The cholinergic MBON-γ2α’1 (dark purple), whose axons project to the superior medial protocerebrum (SMP) and the crepine (CRE), is important for inhibitory control.

Acetylcholine neurons consist of approximately 58% of the central brain^28^ and their axons project to most brain areas (Fig. 2B). The mushroom body (MB) neurons in the central brain are known to modulate locomotor behaviors^29,30^ and their three substructures, namely α/β, α’/β’ and γ neurons, are cholinergic^28^. We asked whether the MB ChAT neurons contribute to the aging-sensitive LIE by using the RNA interference (RNAi)-mediated ChAT knockdown (KD). When ChAT was knocked down in all MB neurons (via the OK107- or MB010B-GAL4 driver), two MB substructures γ and α/β (via MB247- or 201y-GAL4) or γ (via NP1131- or MB009B-GAL4) but not in α/β (via c739- or MB008B-GAL4) or α’/β’ (via c305a- or MB005B-GAL4) neurons led to the aging-dependent increase in LIEs (Fig. 4A, two-way ANOVA: *F*_*32,231*_ = 79.83, *p* < 0.0001; genotype effect, *p* < 0.0001; age effect, *p* < 0.0001; genotype × age, *p* < 0.0001; *n* = 8). These data indicate that the MB γ cholinergic neurotransmission is important for the aging-related LIEs.

Eight types of the mushroom body output neurons (MBON) are known to be cholinergic and their dendrites are mapped to the γ2α’1, α2sc, α2p3p, α3, α’1, α’2, α’3ap and α’3 m lobes^31^. ChAT KD in the MBON-γ2α’1 (via MB077B- or MB051B-GAL4) but not in other cholinergic MBONs, namely MBON-α’2 (via MB018B-GAL4), MBON-α3 (via MB082C-GAL4), MBON-α’1 (via MB543B-GAL4), MBON-α’3ap and -α’3 m (via MB027B-GAL4), MBON-α2sc (via MB549C-GAL4), and MBON-α2p3p (via MB542B-GAL4), resulted in significant LIEs in all ages (4 day, 2 week and 4 week) tested (Fig. 4B, two-way ANOVA: *F*_*26,189*_ = 39.01, *p* < 0.0001; genotype effect, *p* < 0.0001; age effect, *p* = 0.448; genotype × age, *p* = 1; *n* = 8). Thus, the MBON-γ2α’1 cholinergic transmission is critical for inhibitory control. The MBN receive input from the projection neurons (PN) and the PN are cholinergic^20^. We found that ChAT KD in the PN (via NP225- or GH146-GAL4) had no effect on movement inhibition at all ages under test (Fig. 4C, two-way ANOVA: *F*_*8,63*_ = 22.33, *p* < 0.0001; genotype effect, *p* = 0.94; age effect, *p* = 0.0001; genotype × age, *p* = 1; *n* = 8). This suggests that the PN cholinergic transmission is dispensable for inhibitory control.

## Discussion

Inhibition or inhibitory control is a fundamental brain function and is critical for fitness and survival. Poor inhibition is associated with many brain disorders including attention deficit hyperactivity disorder, autism spectrum disorder, addiction and neurodegenerative disorders such as Alzheimer’s disease and frontotemporal dementia^32–36^. Mechanistic studies of inhibition have been done on neurodevelopmental disorders and addiction whereas limited studies have been reported on neurodegenerative dementias. In the report, we show that inhibition capacity declines with aging in wild-type flies, which is halted by the mutation in the acetylcholine breakdown enzyme Ace and augmented by the decreased acetylcholine biosynthesis in the γ MB neurons.

Motor behaviors have been studied in *Drosophila* and they typically entail startle responses to olfactory, visual or mechanosensory stimuli in which increases in locomotor activity or movements represent the expected behavioral outcome^37–40^. In the startle-induced negative-geotaxis/climbing assay, for example, flies exhibit rapid climbing movements when tapped down. Older flies in this assay exhibit slower or less negative-geotaxis movements than young flies^41,42^. The GNG test used in our study measures movement suppression (no or decreased movements) in the presence of strong airflow or predator sound (unpublished data), and we show that aging causes poor suppression represented by hyperkinetic or flying behavior. Reduced or lack of motor activity of aged flies in the startle-induced negative-geotaxis assay could be confounded by general slowness of behavior related to weakened muscle or other physiological changes. The GNG test is beneficial to uncover aging-related changes in neural control of movements, in particular those leading to impulsive acts.

The genetic mutation in the rate-limiting enzyme for acetylcholine biosynthesis ChAT in mice causes lethality at birth^43^ but all mutant mice defective in the individual subunits of nAChR and muscarinic acetylcholine receptor (mAChR) except for α3 are viable to adulthood^44^. The mice lacking the α7 nAChR subunit or the mice with the conditional knockout of M4 mAChR in the D1 receptor expressing neurons^45,46^ show significant increases in impulsive acts, supporting our finding on the acetylcholine’s role in inhibitory control. The genetic knockout studies are very insightful however no human diseases identified to date involves total absence of acetylcholine production nor signaling. It is conceivable that acetylcholine signaling via α7 nAChR and M4 mAChR may decline with aging, which in turn contributes to aging-associated inhibitory control dysfunction. The role of acetylcholine signaling in aging is unknown in *Drosophila*. The only related studies are those demonstrating either reduced or overexpressed vesicular acetylcholine transporter dramatically reduces lifespan of flies, suggesting that acetylcholine is important for longevity^47,48^. Our approach to employ the heterozygous ChAT mutant offers a useful strategy to identify the molecular and cellular players crucial for inhibitory control that is sensitive to aging, a key risk factor for dementia.

The MB is a key neural site processing olfactory behaviors as well as non-olfactory brain functions^49–52^. Our study points to the cholinergic transmission in the γ MB being responsible for aging-related loss of inhibitory control. We have previously shown that the γ neurons, upon receiving dopaminergic and octopaminergic inputs, form appetitive and aversive memory, modulate courtship motivation and develop behavioral sensitization to the disinhibition effect of ethanol^19,53–55^. The γ independently with α’/β’ neurons are also shown to modulate startle-induced negative geotaxis or climbing behavior in which ectopic activation of their activities inhibits the locomotor reactivity to startle but ectopic inhibition of their synaptic outputs has no effect^30^. Since the ChAT RNAi used in this study would dampen cholinergic output from the γ neurons, the neural controls for startle-induced negative geotaxis and movement suppression in the GNG task are likely independent. The study by Martin et al.^29^ shows that the MB ablation or inhibition of γ and/or α/β synaptic output causes basal hyperactivity. While it seems possible that higher basal activity could be associated with more frequent loss of movement suppression, we do not see significant difference in basal activities of the flies with and without ChAT KD at 4 weeks (data not shown). Thus, the neural control of the stimulus-induced movement suppression is distinct from that of basal activity. Together, our study is the first identifying progressive loss of acetylcholine and inhibitory control with aging in which γ cholinergic neurotransmission plays a key role.

The MB axons convey information to 21 types of MBONs among which 8 types are cholinergic^31^. Our study uncovers only 1 type of the cholinergic MBONs—MBON-γ2α’1 (2 neurons per hemisphere) receiving inputs from the MB γ2 and α’1 lobes and sending outputs to the crepine and the superior medial protocerebrum (Fig. 4D)—contributing to movement suppression, which is further supported by our unpublished study demonstrating that ectopic inhibition of MBON-γ2α’1 via Shi^ts^ leads to loss of inhibitory control. MBON-γ2α’1 activity is shown to be crucial for various aspects of appetitive and aversive memory^31,56–60^, courtship memory^61^, sleep promotion^31,62,63^, fat accumulation^64^, food seeking^65^ and attraction/positive valence^31^. Regarding stimulus-induced motor behaviors, ectopic activation of MBON-γ2α’1 causes inhibition of proboscis extension response to sucrose but ectopic inhibition has no effect^66^. The MBONs important for startle-induced negative geotaxis include the cholinergic MBON-α2sc and α’3^30^ but they are not important for movement suppression in the present study. Together, our study reveals a novel role for MBON-γ2α’1 in sustained inhibitory control per se. It remains to be determined whether this function requires the input from the γ MB lobes or dopamine/octopamine neurons or both, and how this MBON with only 2 neurons in each hemisphere accommodates so many functions.

## Data Availability

All raw data reported and materials used in the study are available upon request from the corresponding authors.
